# Natural and human induced factors influencing the abundance of *Schistosoma* host snails in Zambia

**DOI:** 10.1007/s10661-016-5351-y

**Published:** 2016-05-26

**Authors:** Concillia Monde, Stephen Syampungani, Paul J. van den Brink

**Affiliations:** Department of Aquatic Ecology and Water Quality Management, Wageningen University and Research Centre, P.O. Box 47, 6700 AA Wageningen, The Netherlands; Department of Zoology and Aquatic Sciences, Copperbelt University, P.O. Box 21692, Jambo Drive, Riverside Kitwe, Zambia; Department of Plant and Environmental Sciences, Copperbelt University, P.O Box 21692, Jambo Drive, Riverside Kitwe, Zambia; Alterra, Wageningen University and Research Centre, P.O. Box 47, 6700 AA Wageningen, The Netherlands

**Keywords:** Schistosomiasis, Host snails, Epidemiology, Malacological

## Abstract

Schistosomiasis remains a global public health problem affecting about 240 million people. In Zambia, 2 million are infected while 3 million live with the risk of getting infected. Research and interventions relating to schistosomiasis are mainly linked to disease epidemiology. Malacological and ecological aspects of the disease are superficially understood. Developing effective control measures requires an understanding of interacting environmental and socioeconomic factors of host snails vis-a-vis schistosomiasis. Therefore, the present work involved collecting social and environmental data in a large field study in two zones in Zambia that are different in terms of temperature and rainfall amounts. Social data collected through questionnaires included demographic, educational and knowledge of schistosomiasis disease dynamics. Environmental data included physicochemical factors, aquatic plants and snails. Gender (*P* < 0.001) significantly influences livelihood strategies, while age (*P* = 0.069) and level of education (*P* = 0.086) have a moderate influence in zone I. In zone III, none of these factors (age, *P* = 0.378; gender, *P* = 0.311; education, *P* = 0.553) play a significant role. Environmental parameters explained 43 and 41 % variation in species composition for zones I and III, respectively. Most respondents’ (52 %, 87 %) perception is that there are more cases of bilharzia in hot season than in other seasons (rainy season 23 %, 7 %; cold season 8 %, 0 % and year round 17 %, 6 %) for zone I and zone III, respectively.

**Electronic supplementary material:**

The online version of this article (doi:10.1007/s10661-016-5351-y) contains supplementary material, which is available to authorized users.

## Introduction

Schistosomiasis remains a global health problem in the twenty-first century with an estimated 240 million people in 74 countries infected, of whom 90 % are living in sub-Saharan Africa (Sady et al. 2013). The parasites responsible for schistosomiasis affecting humans belong to the genus *Schistosoma* and are dependent on susceptible water snails for their asexual life stages (Boelee and Madsen 2006). Humans are the primary hosts, and transmission between the two hosts takes place in contaminated water (Ojewole 2004). Schistosomiasis is subject to environmental perturbations such as climatic, ecological, hydrological and socioeconomic factors (Michael et al. 2010). As such, the management of schistosomiasis transmission requires an understanding of these interacting factors (Monde et al. 2015). Currently, advanced methodologies for predicting and mapping the prevalences of disease are increasingly being used (Ahmad et al. 2015). For schistosomiasis, GIS and remote sensing techniques have been applied in different parts of the world including China (Zhang et al. 2013; Zhou et al. 2001) and some African countries (Simoonga et al. 2009). These techniques model disease prevalence based on temperature and vegetation data of the target area (Kristensen et al. 2001). While they provide a convenient way to map large-scale (up to 50 km radius; Brooker 2007) environmental preferences of host snails, they do not model small-scale local environmental factors such as demographic, educational and socioeconomic aspects of affected communities which are significant in transmission dynamics of schistosomiasis (Simoonga et al. 2009). Several survey-based studies indicate strong correlation between socioeconomic factors and prevalence of schistosomiasis in the Americas, Asia, (Gazzinelli et al. 2006; Ximenes et al. 2003; Yi-Xin and Manderson 2005) and Africa (Chandiwana and Woolhouse 1991; Kapito-Tembo et al. 2009). Similarly, many studies link environmental (physical, chemical and biological) characteristics of the aquatic body to presence of *Schistosoma* host snails (Giovanelli et al. 2005a; Ndifon and Ukoli 1989). Environmental factors affect the *Schistosoma* host snails’ ability to utilize the habitat and herewith their survival and reproductive potential (Domenici et al. 2007). This link to ecosystem conditions for host-parasite interactions (Patz et al. 2000) is what results in heterogeneities in abundance at local environmental level. Changes in these conditions, whether natural or human mediated, influence the population dynamics of parasites and their hosts and hence epidemiological aspects of the diseases they cause (Patz et al. 2000; Vora 2008). The heterogeneous and complex nature of these interacting factors (Ojewole 2004; WHO 1998) partly explain why schistosomiasis is still a global public health problem especially in Sub-Saharan African countries like Zambia (Chitsulo et al. 2000).

Like many tropical countries, Zambia has struggled with schistosomiasis for several decades. Out of a population of about 13 million, 2 million are said to be infected with schistosomiasis while 3 million are living in constant threat of infection (ZBCP 2009). In some rural communities, infection rate is as high as 90 % of the population (ZBCP 2009). Knowledge on the prevalence of schistosomiasis in Zambia dates back to 1855 (nineteenth century) during David Livingstone’s excursions (Michelson 1989), but the problem remains unabated in the twenty-first century. Among other challenges, the lack of research on the ecological aspects of schistosomiasis exacerbates the problem (Monde et al. 2015). Historic and current research and interventions relating to schistosomiasis are mainly linked to the epidemiology of the disease (Agnew-Blais et al. 2010; Michelson 1989; Strahan et al. 2012). Malacological and ecological aspects of schistosomiasis are superficially understood, and no snail control interventions have been attempted in Zambia (Siziya and Mushanga 1996). This may be attributed to lack of information on the influence of environmental and socioeconomic factors on the population dynamics of *Bulinus globosus* and *Biomphalaria pfeifferi* vis-à-vis schistosomiasis. Developing an understanding of the influence of environmental and socioeconomic factors on the population dynamics of these host snails vis-à-vis schistosomiasis would greatly contribute towards its control.

Therefore, we examined the influence of environmental and socioeconomic factors on the population dynamics of the host snails vis-à-vis schistosomiasis. We evaluated the following:

1. How the behavioural traits of adjacent local human communities are associated with *Schistosoma* infections
2. How habitat factors affect populations of host snails
3. How seasonal variations influence host snail abundance

## Materials and methods

### Description of the study sites

**Fig. 1:**
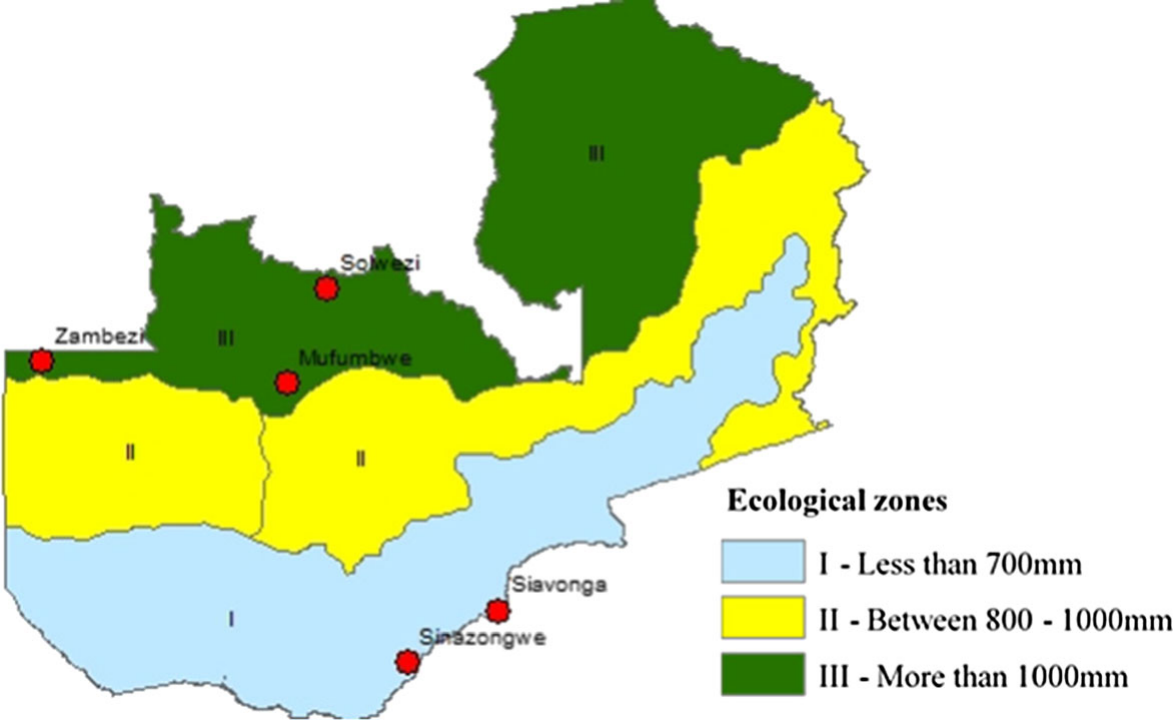
Ecological zones and study sites

The study was conducted in two ecologically distinct zones in Zambia: zone I (Sinazongwe, Siavonga) and zone III (Solwezi, Mufumbwe, Zambezi) (Fig. 1). The two zones receive different amounts of rainfall; zone I receives less than 700 mm while zone III receives between 1000 and 1500 mm annually (MAFF 2001). Zone I is prone to drought, and temperature ranges between 10 and 37 °C. Snail habitat is mainly concentrated in seasonal pools and tributaries of large water systems like Kafue, Zambezi and Kariba systems. Zone III on the other hand is a bit cooler with temperatures ranging between 6 and 32 °C. It is a high rainfall area and has many perennial water systems. Subsistence agriculture (Ndambo 2005) and fishing (Mudenda et al. 2005) are the major livelihood strategies in the study areas. In zone I, small-scale farming (maize) and animal husbandry predominate while small scale-farming (cassava) and fishing are the dominant activities in zone III. Vegetable gardening is an off-season activity common to both zones.

### Experimental design

Data collection was divided into two parts, namely, questionnaire survey and environmental data collection. Each of these surveys was designed differently.

#### Questionnaire survey

The questionnaire survey was designed to collect social data that may have an impact on schistosomiasis dynamics in the study areas. In our survey, all the members of the community who were aged 10 years and above were eligible to participate. The 10-year age cut-off point was thought to be adequate to ensure coherent responses. Verbal permission was sought from all participants or their guardians prior to administering the questionnaires. The questionnaire was pretested by administering it to ten randomly selected community members and adjustments made accordingly. The questionnaire was designed to extract information ranging from demographic (gender, age, livelihood strategy), to educational, to knowledge levels of schistosomiasis disease dynamics. These questionnaires were administered by the researcher and an assistant who served as translator in some cases. A total of 168 (zone I, *n* = 92; zone III, *n* = 76) people participated in this survey.

#### Environmental data

Sampling was done in rivers, streams and dams selected based on clinical records of prevalence of schistosomiasis in adjacent communities as advised by District Health officials (Pers Com.). Sample plots of 10 m × 10 m were established in selected portions of rivers, streams, ox-bow lakes and reservoirs. Where the size of the water body did not allow for establishment of 10 × 10 m plots, the length of the plot was maintained at 10 m while the width was up to the midpoint of the water body. Collection of water samples for determination of physicochemical parameters, estimation for macrophyte cover and snail surveys were done in these plots.

#### Physicochemical properties

Dissolved oxygen, pH, conductivity, turbidity, temperature, nitrate, phosphate and calcium were measured bi-monthly in situ from March 2013 to December 2013 using an AM-200 Aquaread GPS Aquameter. Water and sediment samples for *ex situ* metal analysis were collected into previously rinsed 500-ml polyethylene bottles with polyethylene caps. Each sample was preserved by adding concentrated nitric acid (HNO_3_) to bring the pH to ˃2 (1.5 ml HNO_3_ to 500 ml of sample water) and stored at room temperature. A weak acid extraction method (nitric acid-hydrochloric acid digestion) was used to analyse for metals in water and sediment samples (APHA 1995; Giddings et al. 2001). The flow velocity of water was measured by placing a floating object in the middle of the water body and recording the time taken to drift over a 10-m distance. The substratum was visually categorized as gravel, sandy or muddy at all sites.

#### Aquatic plants

All aquatic plants (macrophytes) were identified based on Cook (2004) and Gerber et al. (2004), while observation based on the established Likert scale of 0 to 5 was used to estimate percent cover of macrophytes over the area. For this scale, 0 indicated no macrophyte while 5 indicated 100 % macrophyte cover. In order to ensure consistency, estimates of macrophyte cover were made by the same person throughout the study.

#### Snails

Two methods were used to collect live snails: by a gloved hand in shallow water and by using a scoop net where water depth could not permit collection by hand. The net was made of 2 × 2-mm mesh size kitchen sieve that was supported by a metal frame mounted on a 1.5-m wooden handle. Search for snails by either way was conducted for 20 min at each site. The search team comprised the same persons all the time. All materials such as boulders, gravel and floating, submerged and emerging vegetation, were searched for snails. Contents of the scoop net were emptied into a tray where snails were identified based on their morphological features (Mandahl-Barth 1962) and counted.

### Data analysis

Two sets of data were analysed, namely, those of the social survey and the environmental and biological data in each zone (zones I and III).

#### Social survey data

Questionnaire responses were coded and entered into SPSS (IBM SPSS Statistics for Windows, Version 22.0) software. Both descriptive and inferential statistics were performed. Descriptive statistics gave an overview of trends in livelihood strategies and water use patterns and were presented as frequencies and percentages. Inferential statistics were performed by multinomial logistic regression with gender, age and educational level as factors. Response variables were livelihood strategies, water use patterns and knowledge levels of disease dynamics.

#### Environmental and biological data

Partial redundancy analysis (RDA) was performed in order to identify factors with the most significant impact on the distribution of the snail community and their correlation. Physical factors including turbidity, colour of water, flow velocity and type of substrate and chemical factors such as pH, nutrients (nitrates, phosphates), heavy metals (copper, cobalt, chromium, cadmium, lead, nickel) dissolved oxygen, oxygen reduction potential, total dissolved solids and electric conductivity were used as predictor variables for snail abundance and distribution. The significance of these factors in explaining the distribution of the snails was determined using permutation tests under the RDA option, using the sampling months as co-variables. A partial RDA was performed which only included the significant explanatory variables (*P* < 0.10) and the sampling months as co-variables. Analyses were performed separately for the samples taken from zones I and III. The RDA analyses were performed with the Canoco version 5 program (Ter Braak and Šmilauer 2012).

To identify environmental factors (Table 1) that act as habitat filters which influence distribution and abundance of aquatic snails (including *Schistosoma* host snails) and to explore how these effects can be quantified, biological and physicochemical parameters were linked to observed densities of aquatic snails.

**Table 1:** Environmental variables monitored in the field for determination of possible impact on abundance and composition of freshwater snails in Zambia

Explanatory variables		Response variables
System attribute	Habitat filter	Molluscs
Chemical properties	pH, nutrients (nitrates, phosphates), heavy metals (Cu, Co, Cr, Cd, Pb, Ni)	Species of freshwater molluscs (composition and abundance)
Physical properties	DO, ORP, TDS, EC	
	Turbidity, colour, flow	
Substrate	Gravel, sandy, muddy, detritus	
Ther	Temperature fluctuations	
Biological regime	Competitors, predators, macrophytes	

Identification of single variables and combinations of variables with high explanatory potential was achieved by use of a linear regression selection method using RSEARCH procedure in GenStat release 12.1 with sampling date as covariate (Payne 2007). For all variables, simple single linear regressions were performed with composition and log-transformed (Ln(x + 1) abundance of snails as endpoints separately. Each variable was paired with each species (excluding species with very low abundances), and the variables with significant correlation coefficients were selected for forward multiple regression. Two levels of significance were adopted: (i) the 5 % probability for type I error (*P* ≤ 0.05) and (ii) the 10 % accepted error probability (0.05 < *P* ≤ 0.1) due to possible high levels of variation in sampling sites.

## Results

### Water contact patterns

**Fig. 2:**
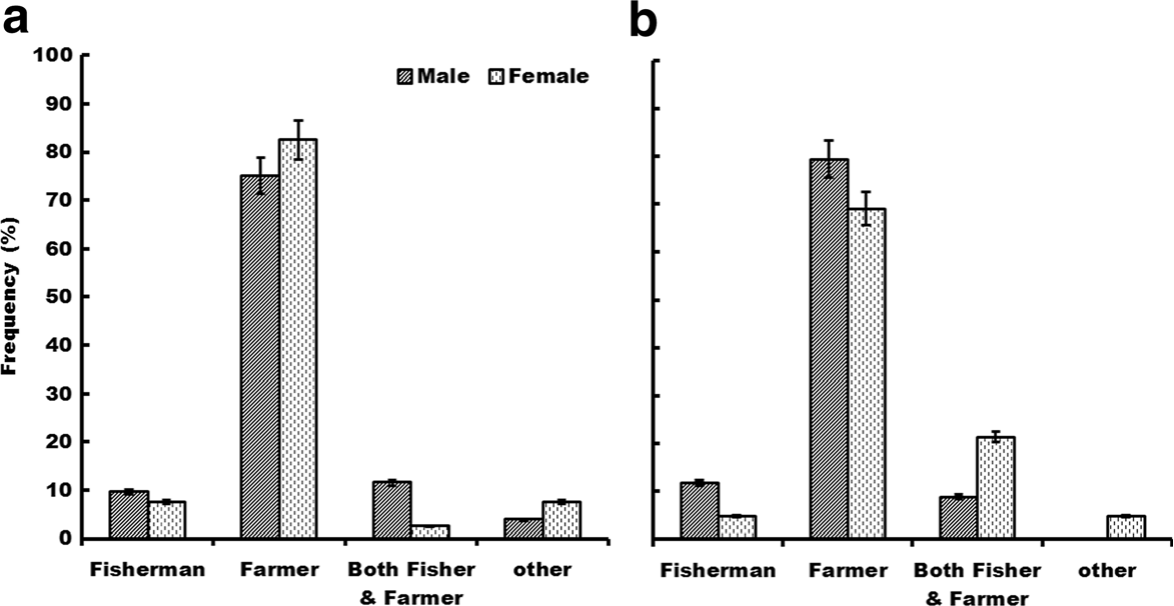
Livelihood strategies across gender in zone I (**a**) and zone III (**b**)

Livelihood strategies are significantly (*P* < 0.001) influenced by gender in zone I but not in zone III (Fig. 2, Table 2). While more women tend to be small-scale farmers, men combine farming and fishing to sustain their families in zone I. Age (*P* = 0.069) and level of education (*P* = 0.086) have a moderate influence on the livelihood strategies in zone I. More people with primary education of ages between 20 and 30 years depend on farming for their livelihood instead of fishing. In zone III, however, none of these factors (age, *P* = 0.378; gender, *P* = 0.311; education, *P* = 0.553) play a significant role.

**Table 2:** Results of multinomial logistic regression analysis

Response variable	Predictor variables	*P* value	
		Zone I	Zone III
Livelihood strategy	Age group	0.069**	0.378
	Gender	<0.001*	0.311
	Level of education	0.086**	0.553
Pattern of river/stream/dam water use	Age-group	0.219	<0.001*
	Gender	0.001*	0.013*
	Level of education	0.079**	0.538
Knowledge of problems due to river/stream/dam water use.	Age group	0.442	0.185
	Gender	0.632	0.075**
	Level of education	0.040*	0.357

Significant *P* values are indicated by an asterisk (*), moderately significant ones by two asterisks (**)

Overall, subsistence farming is the main livelihood strategy in zones I and III. Fishing which is often the next option for rural communities is dominated by men relative to women in zone I and zone III (Fig. 2).

**Fig. 3:**
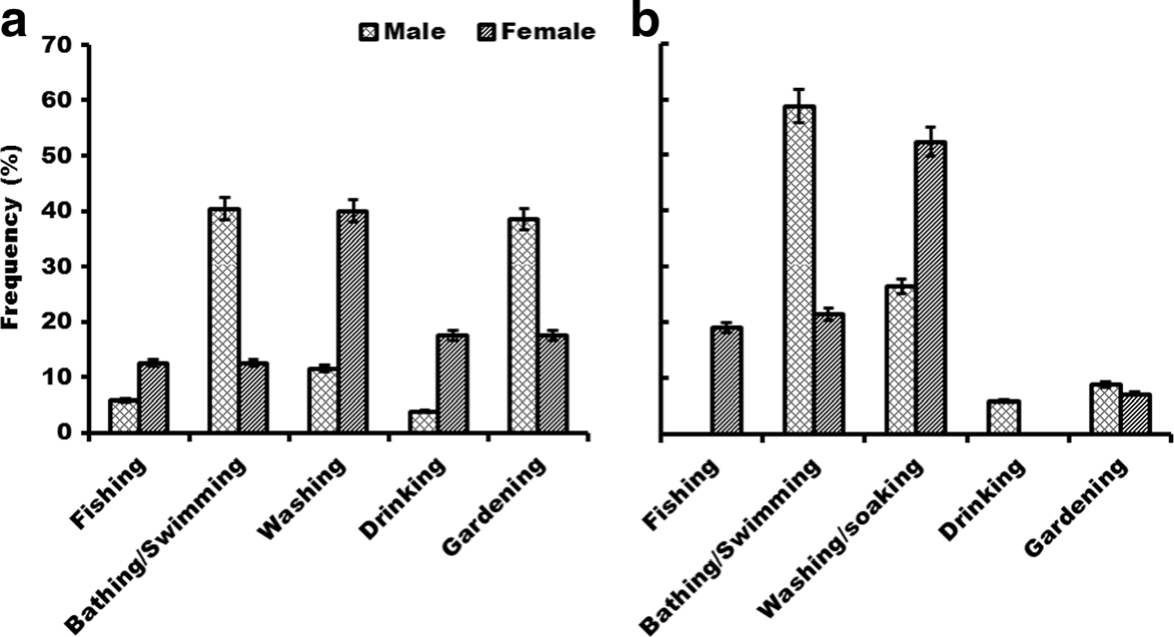
Stream water use across gender in zone I (**a**) and zone III (**b**)

Bathing/swimming, gardening, washing and cassava soaking are the main avenues through which members of the study communities get in contact with water possibly contaminated with *Schistosoma* (Fig. 3). These patterns are gender dependant in both zones (*P* ≤ 0.001, zone I; *P* = 0.013, zone III) (Table 2). More women than men access water contact points during domestic chores like washing plates and clothes and soaking cassava for processing into cassava mill. On the other hand, more men than women are exposed through recreational activities such as bathing and swimming and occupational activities such as gardening (Fig. 3). Level of education moderately influences stream water use patterns in zone I with less educated people (primary level) having more contact with stream water. In zone III, age significantly affects how people get exposed to possibly *Schistosoma*-contaminated water (Table 2). Younger people (16–20) are more likely to get exposed through recreational activities and middle-aged people (26–30) through domestic chores.

Levels of education significantly influenced the knowledge about the *Schistosoma* problem resulting from use of water obtained from streams and dams in zone I (Table 2). Most respondents with some level of education (primary to tertiary) knew that contact with stream/dam water resulted in many health-related problems including schistosomiasis. In zone III, on the other hand, gender has a moderate influence on knowledge levels regarding schistosomiasis (Table 2). Females are comparatively more aware of waterborne diseases than are males. Overall, in zone I, most of the respondents associate bilharzia to contact with stream or dam water followed by diarrhoea, rush and malaria. Similarly, in zone III, contact with stream/dam water is associated by most people with bilharzia, followed by rush, diarrhoea, worms and malaria (Fig. 3). Season is perceived to influence the prevalence of schistosomiasis among the community members. Most of the respondents’ (52 %, 87 %) perception is that the hot season has more cases of bilharzia in the communities than in all other seasons (rainy season 23 %, 7 %; cold season 8 %, 0 % and year round 17 %, 6 %) for zone I and zone III, respectively.

### Environmental parameters and snail abundance

**Fig. 4:**
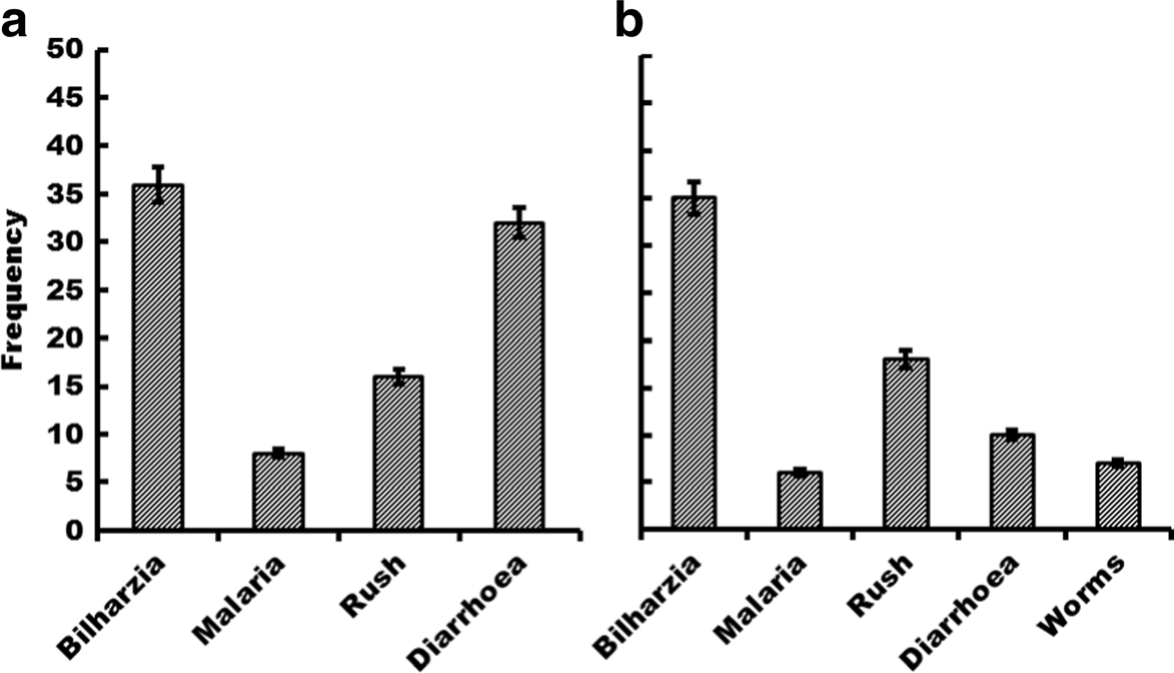
Participants’ perceived problems associated with contact with contaminated water in zone I (**a**) and zone III (**b**)

Five snail species were collected during four repetitive sampling events in both zones I and III: four from class pulmonata (*Lymnaea natalensis, B. globosus, B. pfeifferi, Physa acuta*) and one from class prosobranchia (*Melanoides tuberculata*). Three (i.e. *L. natalensis, B. globosus, B. pfeifferi*) of the four pulmonate snail species are of medical and veterinary importance. In both zones, *M. tuberculata* showed the highest density of 93 % in zone I and 42 % in zone III over the whole period, though with a somewhat erratic distribution over the samples especially in zone I (Fig. 4).

In zone I, *L. natalensis* accounted for 4 % with the highest numbers recorded at the onset of the hot dry season and declining markedly towards the end of the season. *B. globosus* accounted for 2 % while *B. pfeifferi* accounted for 1 % (Fig. 4). *P. acuta* was the least abundant with *n* = 1. The pulmonates *B. globosus* and *L. natalensis* generally showed a similar trend with peaks during the hot dry season (August to November). *B. pfeifferi* was abundant during the hot months of the year.

**Fig. 5:**
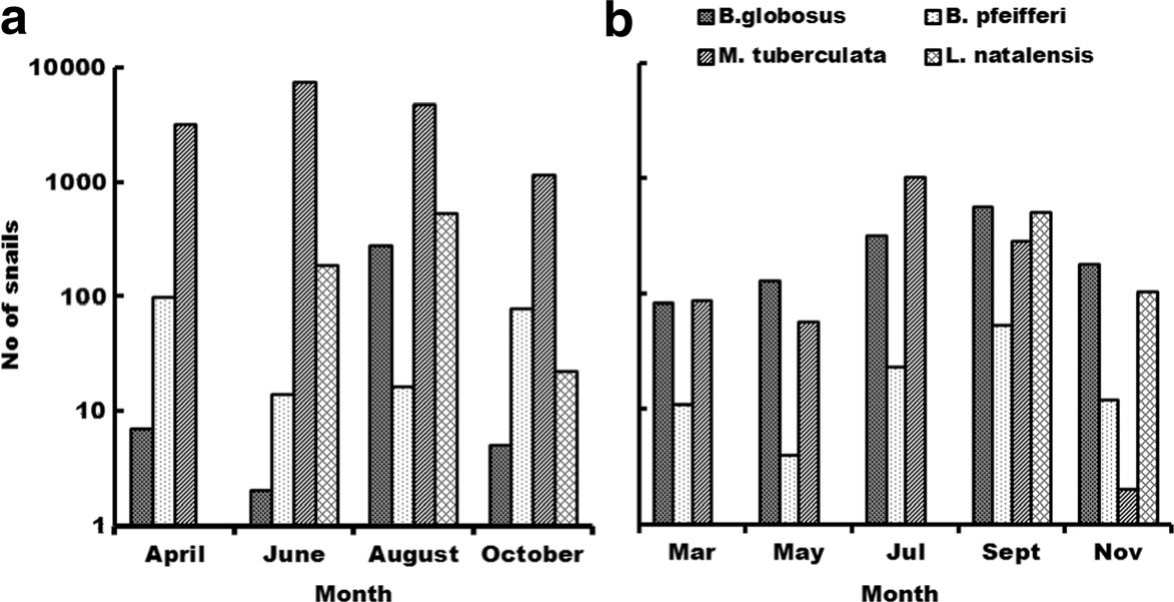
Total number of snails collected over time in zone I (**a**) and zone III (**b**). NB: *P. acuta* not included in the diagram as it was only found once

In zone III, *M. tuberculata* was followed by *B. globosus* with 37 %. *P. acuta* was the least abundant with *n* = 1, while *B. pfeifferi* and *L. natalensis* accounted for 3 and 18 %, respectively. The pulmonates *B. globosus* and *B. pfeifferi* showed a similar trend with peaks during the hot dry season (August to November). Prosobranch *M. tuberculata* on the other hand was present in sufficient numbers during four sampling events but peaked in the cool dry season (April to August) in both zones (Fig. 5).

#### Redundancy analysis

**Fig. 6:**
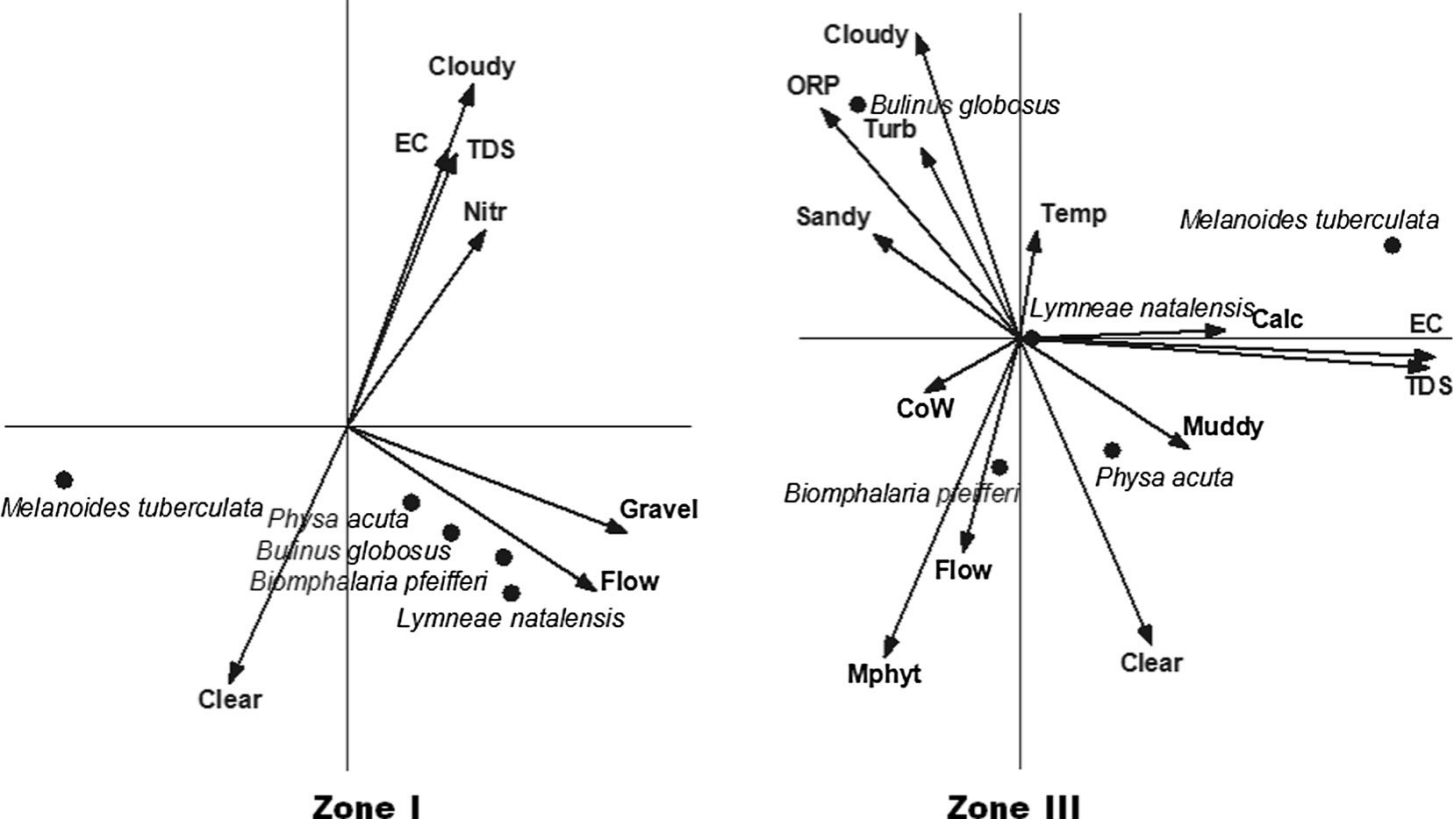
Partial RDA biplot showing snail species found in the study sites and their correlation with environmental parameters of zone I and zone III. For zone I, the explanatory variables explained 43 % of the variation in species composition, while the first and second axes display 82 and 14 % of this variation, respectively. For zone III, the explanatory variables explained 41 % of the variation in species composition, while the first and second axes display 52 and 21 % of this variation, respectively. Key: *Nitr* nitrates, *EC* electric conductivity, *TDS* total dissolved solids, *CoW* cobalt in water, *Calc* calcium, *ORP* oxygen reduction potential, *Temp* temperature, *Turb* turbidity, *MPhyt* macrophytes

The results presented in the RDA biplot (Fig. 6) show that all pulmonate snails recorded are positively correlated with gravel as a substrate and flow velocity in zone I. Prosobranch *M. tuberculata* is positively correlated with clear water and negatively correlated with cloudy water, electric conductivity (EC), total dissolved solids (TDS) and nitrates. *P. acuta* was only found once in both zones, while in zone III, the results show marked variability in habitat selection by the pulmonate snails recorded. *B. globosus* is positively correlated with sandy substrate, ORP and turbid water while *B. pfeifferi* is positively correlated with flow velocity, macrophytes and cobalt. M*. tuberculata* is positively correlated with EC, TDS and to a lesser extent calcium (Fig. 6).

#### Linear regression

Table 3 gives results of the single linear regressions between environmental parameters and snail populations for zones I and III. Parameters with significant (*P* ≤ 0.05) correlations were selected for multiple regression analysis in order to come up with factor combinations with the best explanatory power for the distribution and abundance of snails. The positive (+) and negative (−) signs beside the Adj *R*^2^ values represent the direction of relationship.

**Table 3:** Results of single linear regression where each habitat factor is regressed against each snail species showing *P* values and adjusted *R* ^2^ for zones I and III

*B. globosus*			*B. pfeifferi*			*M. tuberculata*			*L. natalensis*		
Predictor	*P* value	Adj. *R* ^2^	Predictor	*P* value	Adj. *R* ^2^	Predictor	*P* value	Adj. *R* ^2^	Predictor	*P* value	Adj. *R* ^2^
Zone I											
Cd-W	0.001	+29.0	Flow	0.001	+18.9	Gravel	0.001	− 17.1	Cd-W	0.002	+18.1
Flow	0.019	+21.3	Pb-W	0.003	+14.9	Flow	0.006	−12.1	Gravel	0.004	+15.8
Cd-S	0.024	+20.6	Ni-W	0.011	+10.5	Nitr	0.017	−8.6	Flow	0.006	+14.7
Phos-S	0.027	−20.3	Gravel	0.016	+9.5	Cloudy	0.018	−8.2	Cd-S	0.027	+10.1
Chloro	0.056	+18.2	DO	0.037	+6.6	TDS	0.033	−6.3	Chloro	0.075	+6.8
Gravel	0.082	+17.1	Muddy	0.057	−5.2	EC	0.041	−5.6	Cloudy	0.097	−6.0
			Co-S	0.064	−4.8	Clear	0.042	+5.5			
			Calc	0.094	+3.6						
Zone III											
ORP	0.006	+17.3	Flow	0	+19.4	EC	0	+32.7	Co-W	0.002	−47.6
Cloudy	0.006	+17.1	Clear	0.003	+8.4	TDS	0	+31.2	Turb	0.042	−42.2
Clear	0.008	−16.6	Cloudy	0.006	−6.7	Calc	0.002	+9.0	Temp	0.054	+41.9
TDS	0.037	−12.8	Muddy	0.025	+2.8	Mphyt	0.019	−4.2	DO	0.069	+41.5
EC	0.038	−12.7	Co-W	0.04	+1.7	Muddy	0.08	+0			
Turb	0.06	+11.6	Turb	0.054	−0.9	ORP	0.09	−0			
Gravel	0.06	+11.6	Sandy	0.06	−0.6						
Mphyt	0.08	−10.9									
mal Regime											

*Cloudy, clear, turb* condition of water, *Gravel, muddy, sandy* substrate type, *Mphyt* macrophytes, *Co-W, Pb-W, Ni-W* metals in water, *Cd-S, Co-S* metals in sediment, *Flow* flow velocity, *Nitr* nitrates, *Phos* phosphates, *Chloro* clorophyll-a

#### *Multiple regression* (factor combinations)

For zone I, seven significant combinations of two parameters were found for *B. globosus*. The combination with the highest explanatory potential was that of metal content (Cd-W) and flow velocity explaining 38 % (Adj *R*^2^) of the variance in the population dynamics of *B. globosus*, followed by another two-way combination of flow velocity and chlorophyll content explaining 32 % (Adj *R*^2^) of the variance. For *B. Pfeifferi*, 17 combinations were found: ten two-way and seven three-way. The highest variance (Adj *R*^2^ = 41 %) was explained by a three-way combination of flow velocity and two metals (Co-S and Pb-W). Metal content in combination with other factors including type of substrate and levels of dissolved oxygen explain an amount of variance ranging from 32 to 39 %. For *M. tuberculata*, 12 factor combinations were found: eight two-way and four three-way. The three-way factor combination of condition of water (cloudy) flow velocity and type of substrate (gravel) explain 39 % (Adj *R*^2^) of the variance, while a two-way combination of flow velocity and condition of water (cloudy) explain 33 % (Adj *R*^2^) of the variance. In case of *L. natalensis*, nine combinations were possible: seven two-way and two three-way combinations. The highest variance 32 % (Adj *R*^2^) was obtained from a two-way factor combination of metal content (Cd-W) and flow velocity. The highest three-way combination (Adj *R*^2^ = 30 %) was that of chlorophyll-a, flow velocity and type of substrate (gravel).

For zone III, nine factor combinations were found for *B. globosus*. Of these, seven were two-way and two were three-way combinations. The highest explanatory potential was from a three-way combination of condition of water (cloudy), oxygen reduction potential (ORP) and type of substrate (gravel) explaining 28 % (Adj *R*^2^) of the variance in the population dynamics of *B. globosus*. The second best combination with explanatory potential of 25 % (Adj *R*^2^) of the variance was a two-way combination of condition of water (cloudy) and ORP. For *B. pfeifferi*, eight combinations were found: six two-way and two three-way. Similarly, the highest variance Adj *R*^2^ = 28.4 was explained by a three-way combination of flow velocity, metal content (Co) and type of substrate (muddy). For *M. tuberculata* and *L. natalensis*, only two-way combinations were observed to have significant explanatory potential. Electric conductivity and macrophytes explain 43 % (Adj *R*^2^) while metal (Co) content and turbidity explain 50 % (Adj *R*^2^) of the variance in *M. tuberculata* and *L. natalensis,* respectively.

## Discussion

### Water contact patterns

The exposure patterns to *Schistosoma* infection in the study sites include those through domestic, recreational and occupational activities (Figs. 2 and 3). Such exposure patterns were also observed by various researchers in other parts of the world (Fenwick et al. 2006; King and Dangerfield-Cha 2008). However, heterogeneities occur at smaller units such as village groupings and have important implications to the transmission dynamics of schistosomiasis. In Kenya (Satayathum et al. 2006) and China (Liang et al. 2007), differences in infection risks and rates were observed within climatologically homogeneous regions. This heterogeneity was attributed to differences in land use, age and gender structure and kinship at the village level (Satayathum et al. 2006). Our study also confirms the existence of differences in potential transmission pathways based on gender, age and level of education (Table 2). The influence of gender on water use patterns was found to be significant in both zone I and zone III, with the differences being the same for the bathing/swimming and washing activities, but not for the others (Fig. 3). In both zones, recreation activities of bathing and swimming are important exposure pathways for males, while for females, domestic chores including washing of clothes and utensils and soaking of cassava expose them to contaminated water. Similarly, the role of gender in exposure patterns was also reported in Malawi with boys having a higher infection rate than girls due to exposure through swimming and bathing (Kapito-Tembo et al. 2009), in Senegal where females spent more frequent and longer times exposed to contaminated water than males through domestic chores (Sow et al. 2011) and in Zimbabwe with males having higher exposure through recreation activities than women (Chandiwana and Woolhouse 1991). Age is another important factor influencing exposure patterns. In this study, the effect of age on exposure was significant (*P* < 0.001) in zone III but not in zone I. Adolescents and young adults (16–25 years) are more likely to swim and/or bath in streams and other open water areas while older people get exposed through domestic and occupational activities. Similarly in Brazil, Gazzinelli et al. (2006) found a positive correlation between age and prevalence of schistosomiasis, and adolescents and young adults (15–29 years) were most affected. Education is an important tool which governs individual behaviour. In this study, education had a significant (*P* = 0.040) positive correlation with knowledge levels of schistosomiasis in zone I but not in zone III. Educated people are more likely to avoid behaviours that put them at risk of infection. Yang et al. (2015) concluded that “at the individual level, information, education and communication is an essential measure to decrease human exposure to water potentially containing cercariae”. The significant impact of education in zone I can be attributed to the work of the Zambia Bilharzia Control Programme (ZBCP) which has prioritized two provinces (southern and eastern provinces). Although the ZBCP’s main focus is on acquiring and correctly administering praziquantel, health education using information, education and communication materials is an important element of the programme (Kabatereine et al. 2006). These differences in factors influencing exposure patterns in this study confirm the heterogeneous nature of disease patterns which may also reflect the cultural differences among communities (Huang and Manderson 1992). In zone I, for instance, where cattle is a major part of the culture, cattle herding is a risk factor as it involves wading in potentially infested water and is a male activity. This claim is in agreement with Yi-Xin and Manderson (2005) who observed that livestock breeding increases human-water contact activities when they allow their animals to graze near water. In zone III, on the other hand, soaking of cassava for processing into cassava powder, a staple food, involves exposure to potential transmission sites over a considerable time for females. These cultural differences could model the risk of disease in a community between genders.

### Environmental parameters and snail abundance

From the analysis of habitat parameters in both zones, one apparent conclusion is that aquatic snails including hosts of important parasites can tolerate wide variations in their habitat. Therefore, based on these results, it has not been possible to identify a single environmental factor that is a major determinant of host snail population dynamics. Single parameters explained on average a maximum amount of 25 % (min–max, 17–48 %) of the difference in occurrence of the snail species (Table 3). Analysis of various factor combinations yielding an explanatory potential of maximally 50 % indicate that many other factors than investigated in this study interact to condition the habitat for host snails. (Utzinger et al. 1997) and literature cited there in allude to the fact that it is difficult to isolate a single environmental factor as a major determinant for the distribution of host snails. However, in the present study, pulmonate snails in zone I seem to have similar habitat requirements with substrate type and flow velocity being important habitat conditioning parameters (Fig. 6). On the contrary, pulmonates *B. globosus* and *B. pfeifferi* are not influenced by the same factors in zone III (Fig. 6). Differences in ecological and climatic conditions between the zones may be responsible for this result. Zone I is a low rainfall area with mostly disconnected water bodies for most of the year. Water movement in these systems is through wave action and was found to be on average 0.01 ms^−1^. In zone III, a high rainfall area, the water systems are connected for most of the year with an average flow velocity of 0.03 ms^−1^. Flow velocity affects snail movement and dissipation of snail food (Boelee and Laamrani 2004). Some researchers (Appleton 1978) suggested an upper limit for snail prevalence of 0.3 ms^−1^ which is above the flow regime for the study sites. Heavy metal Co is positively correlated with *B. pfeifferi* snails in zone III. Although many metals are known to be nutrients for aquatic organisms at low concentration (Léopold et al. 2015), Pb and Cd have no known biological function and are generally toxic to most organisms (Allah et al. 1997). The positive correlation observed in this study could be as a result of the indirect effects of metals on snails through negatively impacting on the snail parasites and predators. Parasites, like predators, may have a regulatory effect on the snails (Brown et al. 1988; Loker et al. 1981). In a study of populations of two closely related pulmonate snails *Physella columbiana* and *Lymnaea palustris*, it was found that heavy metal pollution had a direct negative effect on parasites that resulted in an indirect positive effect on snails (Lefcort et al. 2002). While metals can result in reduced growth rate, reproduction and survival (Allah et al. 1997; Factor and de Chavez 2012) in snail hosts, it is hypothesized in the present study that metals could have had a more depressing effect on snail parasites and/or predators.

### Seasonal variations

Although population numbers were not rigorously estimated, it was observed that all of the species of medical and veterinary importance exhibited a seasonal trend (Fig. 5). Snail distribution follows a rhythm in response to seasonal climatic variations (Phiri et al. 2007). Temperature and rainfall are important climatic determinants of snail populations (Appleton 1978). In the present study, both *Schistosoma* host snail populations tended to peak around the hot dry season (August to November). Similar results were observed in Zimbabwe (Woolhouse 1992) for *B. pfeifferi*. A lot of *B. globosus* juveniles were observed during this period unlike for *B. pfeifferi* where mostly juveniles were recorded throughout the study period. Other studies indicate the role of temperature on growth rate, reproductive success (El-Emam and Madsen 1982; Woolhouse and Chandiwana 1989) and survival (McCreesh and Booth 2014). Water flow velocity is a seasonal factor regulated by rainfall in Zambia. Impact of rain on stream hydrology and consequent flow velocity and snail prevalence have been observed in Kenya (Teesdale 1962), Tanzania (Marti and Tanner 1988) and Zimbabwe (Woolhouse and Chandiwana 1990; Woolhouse 1992).

## Conclusions

The aim of this study was to provide baseline data on human and environmental factors that influence the prevalence and population dynamics of *Schistosoma* host snails vis-a-vis schistosomiasis transmission in two ecologically distinct zones in Zambia. The focus was twofold covering the social and the physicochemical aspects of host snail population dynamics. We have established from this study firstly that, like in many other studies, rural livelihoods have an impact on the patterning of exposure to schistosomiasis disease. While adhering to the general dynamics of schistosomiasis transmission through domestic, occupational and recreational activities, the study has shown local-level heterogeneities mediated by culture. Cassava processing and cattle herding are culturally determined exposure patterns that vary between the two zones. Second is that, although physicochemical parameters including heavy metals, water flow velocity, type of substrate and condition of water seem to have a significant influence, no single environmental parameter is a major determinant for the distribution of host snails. Third is that climatic conditions associated with season have a profound influence on the prevalence of host snails. Therefore, to address the problem of schistosomiasis requires a delicate balance between disease epidemiology and malacology of the disease vectors. The work being reported in this study forms a baseline in generating social and malacological information which is paramount in addressing schistosomiasis in Zambia. Such information is critical in designing and focussing sustainable control programmes. It highlights habitat conditioning factors including human-water contact patterns that may influence population dynamics of host snails and hence schistosomiasis prevalence. However, the downside of this study is that combining social and environmental aspects did not allow for sufficient in-depth examination of either of these aspects. However, as a baseline study, it has highlighted areas that need further investigation such as the following:

- The breeding patterns of the host snails in these areas. This information can help in planning intervention measures.
- The effect of rainfall on the snail populations. This was not accounted for in this study due to logistical constraints.
- The relationship between pulmonate species and prosobranch *M. tuberculata* because *M. tuberculata* was always found not associated with pulmonate species. This would be important because there are conflicting results from other studies (Giovanelli et al. 2005b; Mkoji et al. 1992; Ndifon and Ukoli 1989; Pointier et al. 1991) regarding the predatory and competitive nature of *M. tuberculata*.

## Electronic supplementary material

Below is the link to the electronic supplementary material.

ESM 1 (DOCX 38 kb)
