# Supplementary material for: Natural and human induced factors influencing the abundance of Schistosoma host snails in Zambia
Source: Environ Monit Assess. 2016 May 26;188:370. doi: 10.1007/s10661-016-5351-y (PMC4882361; doi:10.1007/s10661-016-5351-y)
Supplement: Supplementary file 1 — (DOCX 38 kb) [file 10661_2016_5351_MOESM1_ESM.docx]

Table S1: Zone I multiple regression (Factor combinations)

| **Species 1: B. globosus** | | | | |  | |  |  |  |  | **Species 2: B. pfeifferi** | | | | | | | |  | |  | |  |  |
| --- | --- | --- | --- | --- | --- | --- | --- | --- | --- | --- | --- | --- | --- | --- | --- | --- | --- | --- | --- | --- | --- | --- | --- | --- |
|  |  | |  | |  | |  |  |  |  |  |  | | |  | |  | |  | |  | |  |  |
| **1 predictor** | **p-value** | | **R2 (adj)** | |  | |  |  |  |  | **1 predictor** | **p-value** | | | **R2 (adj)** | |  | |  | |  | |  |  |
| Cdw | 0.001 | | 29.0 | |  | |  |  |  |  | Flow | 0.001 | | | 18.9 | |  | |  | |  | |  |  |
| Flow | 0.019 | | 21.3 | |  | |  |  |  |  | PbW | 0.003 | | | 14.9 | |  | |  | |  | |  |  |
| Cds | 0.024 | | 20.6 | |  | |  |  |  |  | NiW | 0.011 | | | 10.5 | |  | |  | |  | |  |  |
| PhosS | 0.027 | | 20.3 | |  | |  |  |  |  | Gravel | 0.016 | | | 9.5 | |  | |  | |  | |  |  |
| Chloro | 0.056 | | 18.2 | |  | |  |  |  |  | DO | 0.037 | | | 6.6 | |  | |  | |  | |  |  |
| Gravel | 0.082 | | 17.1 | |  | |  |  |  |  | Muddy | 0.057 | | | 5.2 | |  | |  | |  | |  |  |
|  |  | |  | |  | |  |  |  |  | CoS | 0.064 | | | 4.8 | |  | |  | |  | |  |  |
|  |  | |  | |  | |  |  |  |  | Calc | 0.094 | | | 3.6 | |  | |  | |  | |  |  |
| **Multi-predictors (included all parameters with R2 (adj) > 6.5)** | | | | | | | | | |  | **Multi-predictors (included all parameters with R2 (adj) > 0.00)** | | | | | | | | | | | | | |
|  | |  | |  | |  |  |  |  |  |  | | |  |  |  | |  | |  | |  | |  |
| **2 predictors** | |  | |  | |  |  |  |  |  | **2 predictors** | | | |  |  | |  | |  | |  | |  |
| **pred 1** | | **p-value** | | **pred 2** | | **p-value** | **R2 (adj)** |  |  |  | **pred 1** | | **p-value** | | **pred 2** | **p-value** | | **R2 (adj)** | |  | |  | |  |
| Cdw | | 0.000 | | Flow | | 0.006 | 38.4 |  |  |  | Flow | | 0.000 | | PbW | 0.002 | | 32.7 | |  | |  | |  |
| Flow | | 0.002 | | Chloro | | 0.005 | 31.9 |  |  |  | Flow | | 0.001 | | NiW | 0.006 | | 29.2 | |  | |  | |  |
| Flow | | 0.013 | | Cds | | 0.016 | 28.9 |  |  |  | Flow | | 0.001 | | CoS | 0.039 | | 24.3 | |  | |  | |  |
| Flow | | 0.013 | | PhosS | | 0.018 | 28.6 |  |  |  | PbW | | 0.001 | | DO | 0.012 | | 24.0 | |  | |  | |  |
| Cds | | 0.046 | | PhosS | | 0.052 | 25.1 |  |  |  | PbW | | 0.003 | | Gravel | 0.014 | | 23.6 | |  | |  | |  |
| PhosS | | 0.021 | | Gravel | | 0.061 | 24.4 |  |  |  | PbW | | 0.001 | | CoS | 0.017 | | 23.0 | |  | |  | |  |
| Chloro | | 0.038 | | Gravel | | 0.055 | 22.7 |  |  |  | Flow | | 0.001 | | DO | 0.064 | | 23.0 | |  | |  | |  |
|  | |  | |  | |  |  |  |  |  | NiW | | 0.005 | | Gravel | 0.007 | | 21.6 | |  | |  | |  |
|  | |  | |  | |  |  |  |  |  | PbW | | 0.003 | | Muddy | 0.055 | | 19.6 | |  | |  | |  |
|  | |  | |  | |  |  |  |  |  | Gravel | | 0.004 | | CoS | 0.015 | | 18.4 | |  | |  | |  |
|  | |  | |  | |  |  |  |  |  |  | |  | |  |  | |  | |  | |  | |  |
| **3 predictors** | |  | |  | |  |  |  |  |  | **3 predictors** | | | |  |  | |  | |  | |  | |  |
| **pred 1** | | **p-value** | | **pred 2** | | **p-value** | **pred 3** | **p-value** | **R2 (adj)** |  | **pred 1** | | **p-value** | | **pred 2** | **p-value** | | **pred 3** | | **p-value** | | **R2 (adj)** | |  |
|  | |  | |  | |  |  |  |  |  | Flow | | 0 | | PbW | 0 | | CoS | | 0.007 | | 41.2 | |  |
|  | |  | |  | |  |  |  |  |  | Flow | | 0.001 | | PbW | 0.001 | | DO | | 0.019 | | 38.9 | |  |
|  | |  | |  | |  |  |  |  |  | Flow | | 0 | | PbW | 0.015 | | NiW | | 0.06 | | 36.3 | |  |
|  | |  | |  | |  |  |  |  |  | PbW | | 0 | | CoS | 0.002 | | Gravel | | 0.002 | | 36.1 | |  |
|  | |  | |  | |  |  |  |  |  | PbW | | 0.001 | | DO | 0.005 | | Gravel | | 0.005 | | 34.3 | |  |
|  | |  | |  | |  |  |  |  |  | Flow | | 0 | | NiW | 0.01 | | CoS | | 0.058 | | 33.1 | |  |
|  | |  | |  | |  |  |  |  |  | Flow | | 0.001 | | NiW | 0.006 | | DO | | 0.062 | | 32.9 | |  |
|  | |  | |  | |  |  |  |  |  |  | |  | |  |  | |  | |  | |  | |  |
|  | |  | |  | |  |  |  |  |  |  | |  | |  |  | |  | |  | |  | |  |
|  | |  | |  | |  |  |  |  |  |  | |  | |  |  | |  | |  | |  | |  |
|  | |  | |  | |  |  |  |  |  |  | |  | |  |  | |  | |  | |  | |  |

Table S2: Zone III multiple regression (Factor combinations)

| **Species 1: B. globosus** | | | | | | | |  |  | |  | |  | | | | **Species 2: B. pfeifferi** | | | | | | |  |  |  | | | |
| --- | --- | --- | --- | --- | --- | --- | --- | --- | --- | --- | --- | --- | --- | --- | --- | --- | --- | --- | --- | --- | --- | --- | --- | --- | --- | --- | --- | --- | --- |
| **1 predictor** | | | **p-value** | | | **R2 adj)** | |  |  | |  | |  | | | | **1 predictor** | | **p-value** | | **R2 adj)** |  | |  |  |  | | | |
| ORP | | | 0.006 | | | 17.3 | |  |  | |  | |  | | | | Flow | | 0 | | 19.4 |  | |  |  |  | | | |
| Cloudy | | | 0.006 | | | 17.1 | |  |  | |  | |  | | | | Clear | | 0.003 | | 8.4 |  | |  |  |  | | | |
| Clear | | | 0.008 | | | 16.6 | |  |  | |  | |  | | | | Cloudy | | 0.006 | | 6.7 |  | |  |  |  | | | |
| TDS | | | 0.037 | | | 12.8 | |  |  | |  | |  | | | | Muddy | | 0.025 | | 2.8 |  | |  |  |  | | | |
| EC | | | 0.038 | | | 12.7 | |  |  | |  | |  | | | | Cow | | 0.04 | | 1.7 |  | |  |  |  | | | |
| Turb | | | 0.06 | | | 11.6 | |  |  | |  | |  | | | | Turb | | 0.054 | | 0.9 |  | |  |  |  | | | |
| Gravel | | | 0.06 | | | 11.6 | |  |  | |  | |  | | | | Sandy | | 0.06 | | 0.6 |  | |  |  |  | | | |
| Mphyt | | | 0.08 | | | 10.9 | |  |  | |  | |  | | | |  | |  | |  |  | |  |  |  | | | |
| **Multi-predictors (included all parameters with R2 (adj) > 6.5)** | | | | | | | | | | | | | | | | | **Multi-predictors (included all parameters with R2 (adj) > 0.00)** | | | | | | | | | | | | |
| **2 predictors** | | | | | | | | | | | | | | | | | **2 predictors** | | | | | | | | | | | | |
| **pred 1** | **p-value** | | | **pred 2** | | | **p-value** | | | **R2(adj)** | |  | | |  | | **pred 1** | | **p-value** | **pred 2** | | | **p-value** | | **R2 (adj)** | | |  |  |
| ORP | 0.007 | | | Cloudy | | | 0.008 | | | 25.2 | |  | | |  | | Flow | | 0 | Cow | | | 0.033 | | 24 | | |  |  |
| ORP | 0.012 | | | Clear | | | 0.016 | | | 23.7 | |  | | |  | | Flow | | 0.001 | Clear | | | 0.099 | | 21.7 | | |  |  |
| Cloudy | 0.005 | | | Gravel | | | 0.036 | | | 21.7 | |  | | |  | | Clear | | 0.003 | Cow | | | 0.037 | | 13.3 | | |  |  |
| Clear | 0.006 | | | Gravel | | | 0.04 | | | 20.9 | |  | | |  | | Muddy | | 0.005 | Cow | | | 0.007 | | 12.6 | | |  |  |
| ORP | 0.006 | | | Mphyt | | | 0.083 | | | 20 | |  | | |  | | Cow | | 0.007 | Sandy | | | 0.01 | | 10.4 | | |  |  |
| ORP | 0.009 | | | Turb | | | 0.097 | | | 19.7 | |  | | |  | | Cloudy | | 0.011 | Cow | | | 0.066 | | 10.3 | | |  |  |
| Cloudy | 0.003 | | | PhosS | | | 0.097 | | | 19.5 | |  | | |  | |  | |  |  | | |  | |  | | |  |  |
| **3 predictors** | | | | | | | | | | | | | | | | | **3 predictors** | | | | | | | | | | | | |
| **pred 1** | | **p-value** | | | **pred 2** | | | **p-value** | | | **pred 3** | | | **p-value** | | **R2 (adj)** | **pred 1** | **p-value** | | **pred 2** | | | **p-value** | | **pred 3** | | **p-value** | | **R2 (adj)** |
| Cloudy | | 0.005 | | | ORP | | | 0.016 | | | Gravel | | | 0.081 | | 27.7 | Flow | 0 | | Cow | | | 0.009 | | Muddy | | 0.033 | | 28.4 |
| Clear | | 0.011 | | | ORP | | | 0.025 | | | Gravel | | | 0.088 | | 26.1 | Flow | 0.001 | | Cow | | | 0.033 | | Clear | | 0.097 | | 26.3 |
